# Wear Analysis of Materials Used for a Track Steering System in Abrasive Soil Mass

**DOI:** 10.3390/ma14206164

**Published:** 2021-10-18

**Authors:** Klaudia Olejniczak, Jerzy Napiórkowski

**Affiliations:** Department of Construction, Vehicle and Machine Operation, Faculty of Technical Sciences, University of Warmia and Mazury in Olsztyn, M. Oczapowskiego 11, 10-719 Olsztyn, Poland; jerzy.napiorkowski@uwm.edu.pl

**Keywords:** steering system, abrasive wear, Hadfield steel, abrasive soil mass

## Abstract

This paper presents the results of comparative research on materials used for a track steering system in an abrasive soil mass. Two types of elastomer tracks were tested: a steel-rubber stave from an asphalt paver and a rubber overlay used in vehicles with a steel track chain. The results obtained were related to the wear of Hadfield steel. The tests were carried out on a “spinning bowl” stand in a natural soil mass, which consisted of two types of soil: light and heavy. It was shown that the resistance to abrasive wear depended on the grain size of the worked soil and the chemical composition of the materials. Rubber overlay was found to have the highest resistance index in all types of soils. It was made of high-density polyethylene, low-density polyethylene, ethylene acrylate/ethyl copolymer (ethylene acrylate 18%) and ethylene/propylene copolymer with an ethylene content of 60%. An analysis of the condition of the machined surfaces after friction tests complements the results presented.

## 1. Introduction

Due to the varied types of substrate on which a vehicle needs to travel, the traditional wheeled chassis is very often replaced by a track system. Track vehicles can operate very well in swampy, desert, or snowy terrain. This is because the weight of the vehicle is distributed over a much larger area than in a wheeled chassis. This increases the vehicle’s grip, generates more traction force and lowers individual pressures. The track transfers longitudinal, vertical and lateral forces that emerge in contact with the ground. Bęben [1] discussed the significance of the influence of environmental conditions on the values of the coefficient of friction in elastomer tracks. For the same moisture content, the value of the coefficient of friction was 0.9 in clay and 0.3 in the sand. This may be indicative of a different friction behaviour in the soil conditions specified. With regard to the structure, metal, rubber-metal and rubber tracks can be distinguished [2,3]. Among others, Hadfield cast steel is used for the links [4]. According to [5,6], the wear resistance of a metal depends on the grain refinement and the content of carbide needles (FeMn)_3_C, usually eliminated by heat treatment. After heat treatment, typical manganese cast Hadfield steel has an austenitic structure which, despite its low hardness, gives high abrasion resistance compared to other engineering materials [7]. Elastomer is the basic component of rubber tracks, in which polyethylene acts as a filler. Several types can be distinguished, depending on polyethylene density. The most popular types include high-density polyethylene 0.935–0.965 g/cm^3^ (PE-HD) and low density polyethylene 0.918–0.930 g/cm^3^ (PE-LD) [4]. The question thus arises as to whether elastomer tracks exhibit a higher abrasion resistance compared to traditional metal track systems. To date, little information has been provided in the literature on the wear behaviour of materials used in track steering systems [3,8]. The complexity of the polymer wear process, in terms of its chemical composition, type of wear and the wear environment, has been presented, among others, in [9,10,11,12,13,14,15,16,17,18,19]. These papers do not take into account wear interaction with the natural soil mass. It is characterised by peculiar wear properties, which were quite well described in terms of impact on metals [20,21,22,23]. In this analysis, it was found that only a few works discussed the influence of soil mass on polymer wear [24,25]. 

This paper analysed the wear resistance of materials used for a tracked chassis in an abrasive soil mass with reference to Hadfield steel.

## 2. Research Methodology

### 2.1. The Test Procedure

Cuboids sized 30 mm × 25 mm × 10 mm were taken as samples of the tested materials with a method ensuring the invariability of their structure. A high-energy abrasive water jet cutting method was used to cut the samples.

The chemical composition of Hadfield cast steel was determined using a spectral method with a GDS500A glow discharge spectrometer from Leco, using the following parameters: U = 1250 V, I = 45 mA, argon. The arithmetic mean of five measurements was taken as a result. Chemical components were identified in elastomeric materials using infrared spectroscopy (IR) with the SHIMADZU ITRracer-100 apparatus and the reflection method (ATR). This method does not allow the proportions of the components in elastomers to be determined.

The hardness of Hadfield cast steel was measured using the Vicker’s method in conditions compliant with PN-EN ISO 6507-1:1999. Zwick 32 hardness tester, with a load of 1 kg (9.807 N) and operating for 15 s, was used for the measurements. The hardness of elastomeric samples was measured with the Shore method according to PN-ISO 868. The density of the elastomers tested was determined by comparing the mass and volume of the prepared samples.

To implement the research objectives for macroscopic examination and evaluation of surfaces after abrasion tests, a KEYENCE Digital Microscope (VHX-6000 series) was used.

**Figure 1:**
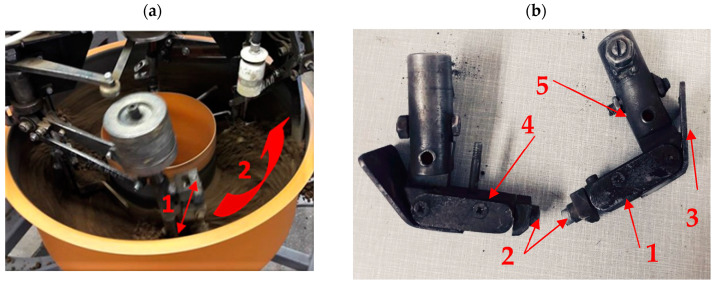
The “spinning bowl” type laboratory wear testing stand: (**a**) fragment of the stand during the operation; arrow (1) shows the direction of the arm movement, (2) shows the direction of the bowl rotation. (**b**) feet mounted on the stand arms with a designated place for fixing the sample; (1) sample fixing place, (2) screws holding the sample, (3) front skid, (4) side sample cover, (5) screw for changing the rake angles.

Wear intensity tests were performed in laboratory conditions using the “spinning bowl” method (Figure 1) [26]. The machine’s bowl was filled with an abrasive soil mass, which successively consisted of two types of soil marked as light (loamy sand) and heavy (ordinary clay). The tests were repeated five times. Each sample travelled a total friction distance of 10,000 m at a speed of 1.66 m/s and a unit pressure of 67 kPa. The mass sample was measured every 2000 m on a laboratory scale with an accuracy of 0.0001 g after cleaning in an ultrasonic cleaner. The samples moved in an oscillatory motion along the friction track. The pH of the abrasive masses ranged from 6.3 to 6.9 pH, and the moisture content ranged from 11% for loamy sand to 14% for ordinary clay, which corresponds to moist soil. Soil moisture content was determined by measuring the weight of the solid phase dried at 105 °C. Grain size analysis was carried out by laser diffraction using a Mastersizer 2000 laser particle composition meter according to ISO 13320 (Table 1).

The mass wear of the sample and its mass wear intensity were determined from the following relationships:

—sample mass wear;

(1) $$Z_{\mathrm{pw}}=m_{w}-m_{i}\;\left[g\right],$$

where:

m_w_—input sample mass before friction [g],
m_i_—sample mass after travelling the friction path S [g],
—mass wear intensity;

(2) $$I_{\mathrm{pw}}=\frac{Z_{\mathrm{pw}}}{S}\;\left[\frac{g}{\mathrm{km}}\right],\;$$

where:

S—friction path [km].

Based on the K_b_ wear resistance index from the formula [10], the wear resistance of the tested materials was compared:

(3) $${\;K}_{b}=\frac{\frac{Z_{\mathrm{Vw}}}{S_{\mathrm{Tw}}}}{\frac{Z_{\mathrm{Vb}}}{S_{\mathrm{Tb}}}}=\frac{Z_{\mathrm{Ww}}\times \rho_{b}\times S_{\mathrm{Tb}}}{Z_{\mathrm{Wb}}\times \rho_{w}\times S_{\mathrm{Tw}}}\;,\;$$

where:

Z_Vw_—volumetric wear of the reference material;
Z_Vb_—volumetric wear of the tested material;
Z_Ww_—mass wear of the reference material;
Z_Wb_—mass wear of the tested material;
S_Tw_—friction path of reference material;
S_Tb_—friction path of tested material;
ρ_w_—density of the reference material;
ρ_b_—density of the tested material.

### 2.2. Test Subject

Elastomer samples were taken from a tractor track, a mini-excavator track, a steel-rubber stave of an asphalt paver, and a rubber boot used on steel track vehicles.

**Figure 2:**
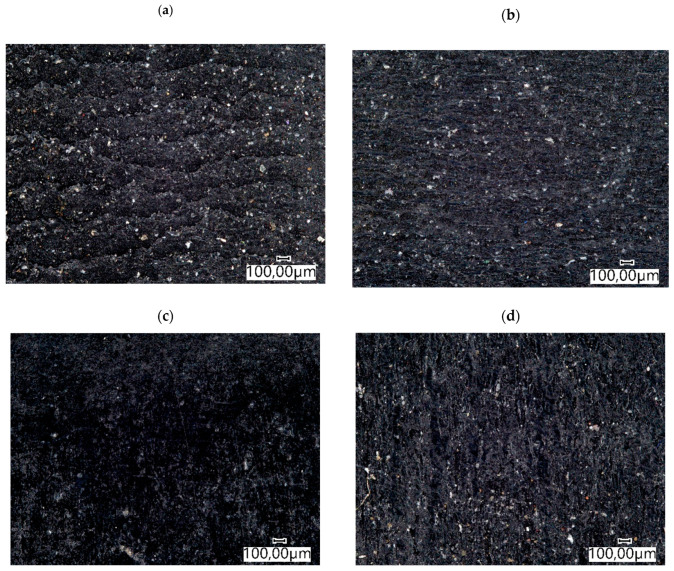
Surface view of the tested elastomers before the wear test: (**a**) track from a tractor; (**b**) track from a mini excavator; (**c**) steel-rubber stave; (**d**) rubber pad.

**Figure 3:**
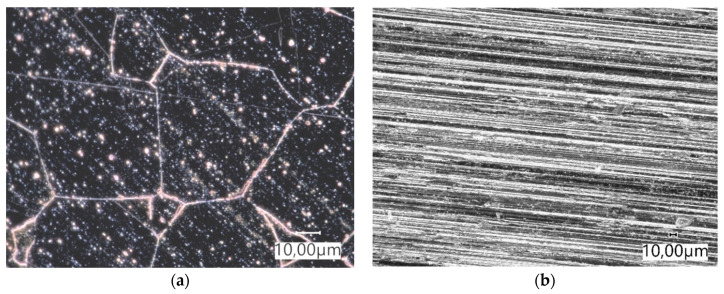
(**a**) Microstructure of Hadfield steel. Coarse-grained structure of unbalanced pearlite with bright ferrite separations at grain boundaries and within grains. Mi1Fe etching, light microscopy. (**b**) surface view of the sample before the tribological test.

In the tractor and the mini-excavator track, samples were cut from seamless elastomeric tracks. The test material was taken from the part of the component located outside the reinforcement. Figure 2 shows the surface view of the elastomers before the tribological test. The obtained wear test results were related to Hadfield steel (Figure 3) (Table 2).

The density of the tested elastomers was determined by the laboratory method based on the comparison of the mass and volume of the prepared samples (Table 3).

**Figure 4:**
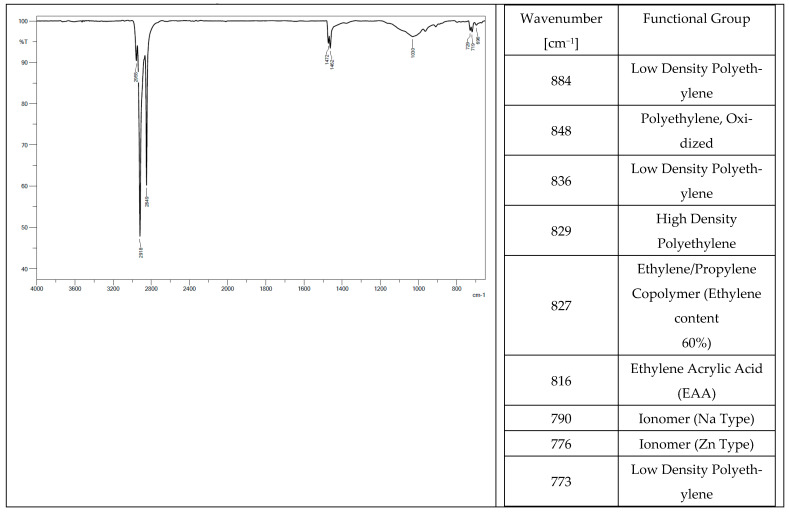
Spectrum and band characteristics obtained with the FT-IR method for a track from the tractor.

**Figure 5:**
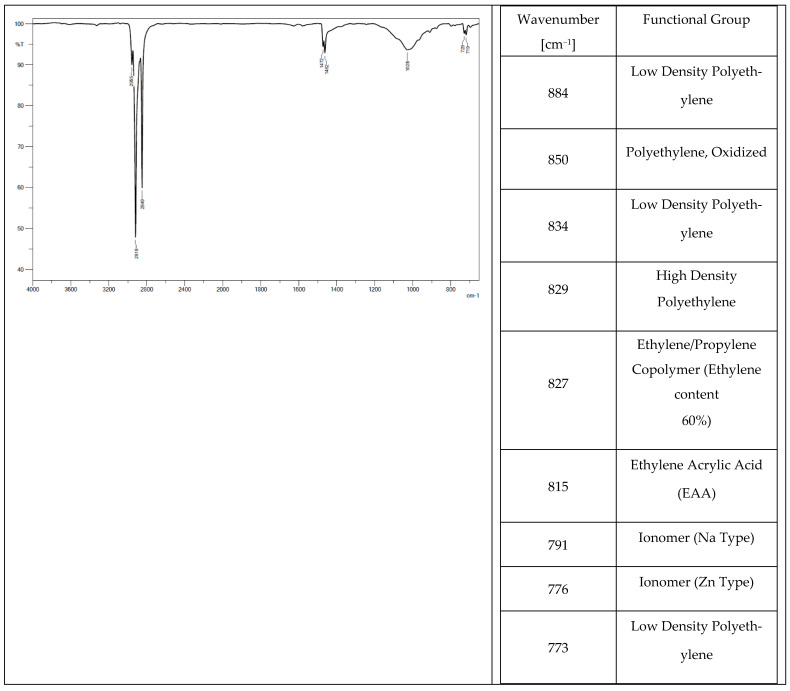
Spectrum and band characteristics obtained with the FT-IR method for a track from the mini-excavator.

**Figure 6:**
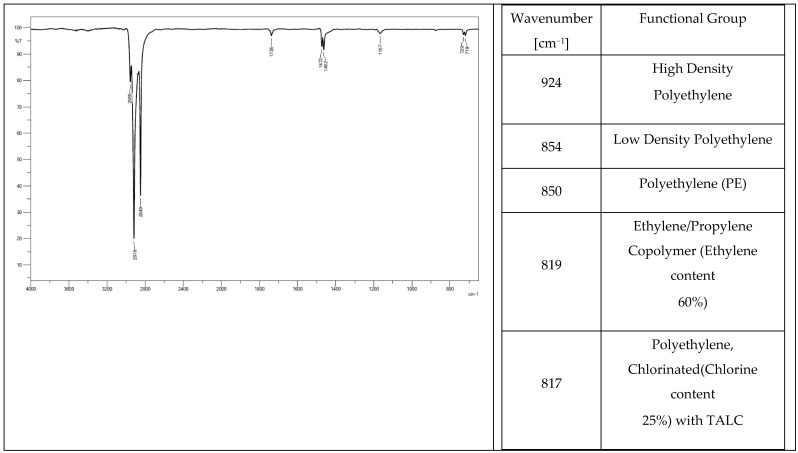
Spectrum and band characteristics obtained with the FT-IR method for a track from the steel-rubber stave.

**Figure 7:**
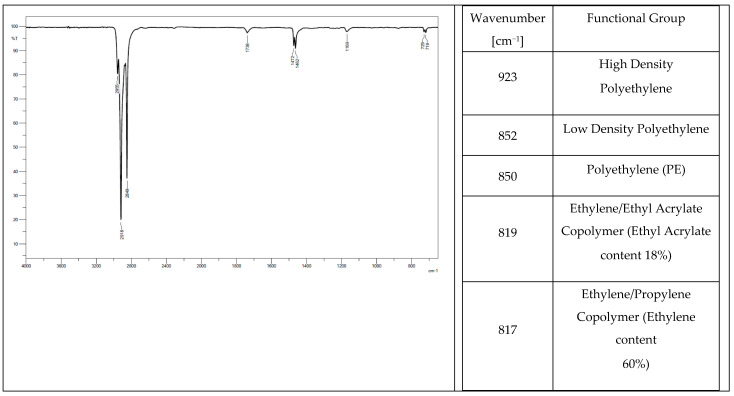
Spectrum and band characteristics obtained with the FT-IR method for the rubber pad.

Low-density polyethylene (PE-LD), ethylene/propylene copolymer with an ethylene content of 60%, high-density polyethylene (PE-HD) were found in the chemical composition of all the tested elastomers. Oxidized polyethylene, ethylene-acrylic acid, and Na- and Zn-type ionomers were additionally identified in tracks from a tractor (Figure 4) and a mini-excavator (Figure 5). Ethylene acrylate/ethyl copolymer (ethyl acrylate content 18%), chlorinated polyethylene (chlorine content 25%) with talc, and polyethylene (PE), which is also present in the rubber pad (Figure 6), were identified in the steel-rubber stave (Figure 7).

Oxidised polyethylene is a component of aqueous emulsions with a wide range of applications, from high abrasion resistance pastes for floor care, to the production of polymeric plastics such as PVC, where it is used as a lubricating additive [26].

Talc Mg_3_(Si_4_O_10_)(OH)_2_ is a crystalline form of magnesium silicate. It is classed as a soft mineral, characterised by the lowest hardness on the Mohs scale. It exhibits antistatic and anti-adhesion properties. Due to its hydrophobic properties, it dissolves well in polyolefins, e.g., polyethylene (PE), and is therefore used as a filler in the production of polymer composite materials based on PE and PP. Because talc is cheaper than typical polymers (PE or PP), it is often used in the production of polymeric products, mainly based on hydrophobic polyolefins. [27].

Chlorinated polyethylene is an elastomer that takes a form of a white powder. It consists of high-density polyethylene and chlorine. The addition of such a compatibilizer affects shear strength or stability in heat treatment. An increase in the modulus of elasticity and strength of composites is observed [28].

In the chemical industry, ethyl acrylate is commonly used as a monomer to obtain various types of polymers, to produce plastics or synthetic rubbers. It is obtained by propylene oxidation. Propylene oxidation results in acrolein, which is then oxidised to acrylic acid. Ethyl acrylate is produced by reacting acrylic acid with ethanol.

Ethylene/propylene copolymer is produced during polymerisation in a solution using Ziegler-Natta catalysts. With double bonds found inside groups, it is much less sensitive to weathering and sunlight than polyethylene.

Low-density polyethylene (PE-LD) is called high-pressure polyethylene because of the way it is produced in the gas phase under high pressure and at high temperatures. In the presence of hydroxides or peroxides as catalysts, the polymerisation process results in a product with the consistency of honey. After passing through a pressure-reducing tank, it takes the form of a ribbon which granulates after cooling. PE-LD is obtained as a result of free radical polymerisation at 150 ÷ 260 °C temperature without solvent, 150 ÷ 200 MPa pressure, and oxygen or organic peroxide as reaction initiator. The maximum oxygen content is 0.5% by volume. Exceeding this limit has an unfavourable effect on the polymer structure. Ethylene meets stringent purity criteria (99.8 ÷ 99.9%) and does not contain impurities such as hydrogen and acetylene. The polymer properties and quality depend not only on the purity of the raw material but also on the parameters, especially temperature.

High-density polyethylene (PE-HD) is obtained at a temperature of 50–70 °C in the liquid phase using Ziegler-Natta catalysts. Compared to the high-pressure method, the apparatus is less complicated, but the use of a large number of solvents, organometallic catalyst, and its leaching from the polymer creates many difficulties and increases the process cost. It is produced under low pressure. In the low-pressure ethylene polymerisation, organometallic catalysts, titanium tetrachloride—TiCl4, and triethylaluminium—Al(C2H5)3, are used to form a complex to catalyse the reaction.

## 3. Analysis of the Results

**Figure 8:**
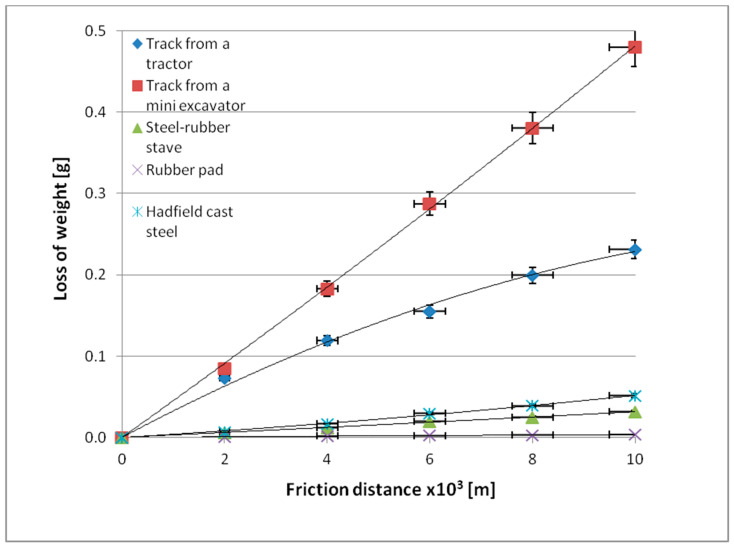
Mass wear as a function of friction path travelled by the tested materials in light soil.

**Figure 9:**
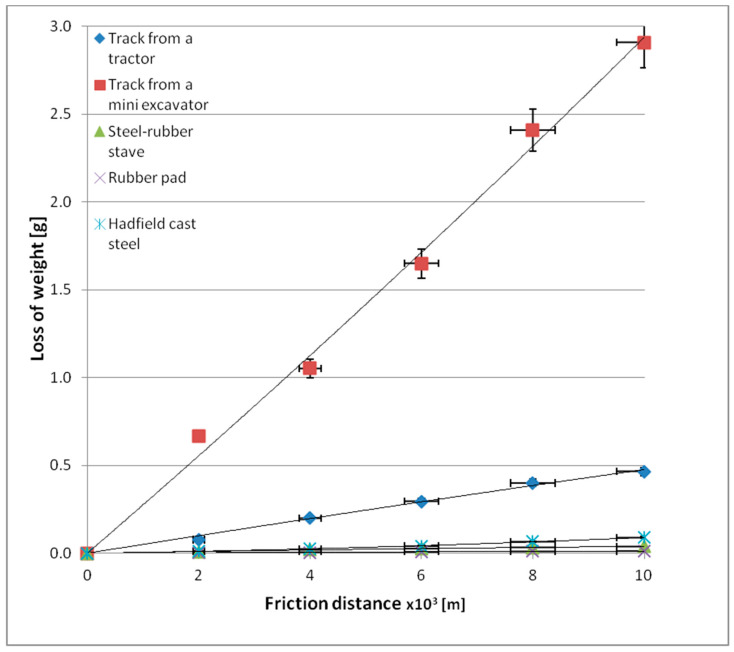
Mass wear as a function of friction path travelled by the tested materials in heavy soil.

Table 4 presents results for mass loss after a distance of 10,000 m, while Figure 8 and Figure 9 show wear behaviour as a function of friction distance under varying soil conditions.

**Figure 10:**
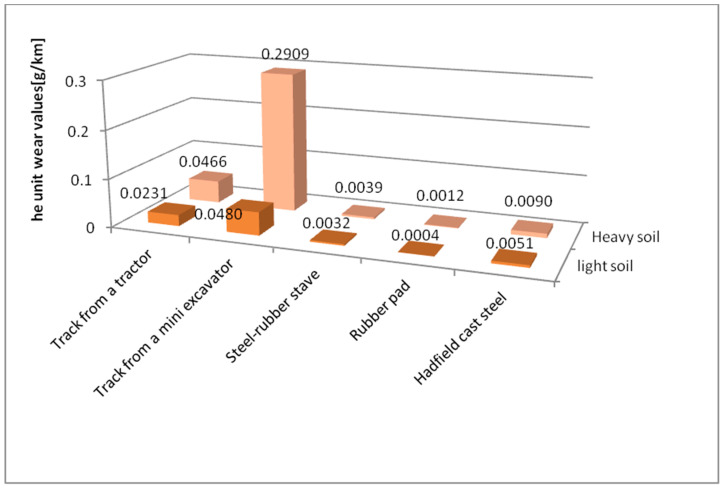
Wear intensity of the tested materials in specific soils.

Mass wear behaviour for the materials accepted for testing as a function of the friction path travelled is described by linear equations (Figure 8 and Figure 9). The wear values for the tested materials change with the change in the soil grain size. The lowest wear values were recorded for the rubber pad composed of high-density polyethylene, low-density polyethylene, ethylene acrylate/ethyl copolymer (ethylene acrylate 18%), and ethylene/propylene copolymer with an ethylene content of 60%, regardless of the soil type. The highest wear values were obtained for a mini-excavator track containing low-density polyethylene, high-density polyethylene, ethylene/propylene copolymer with an ethylene content of 60%, oxidised polyethylene, ethylene-acrylic acid, and Na and Zn type ionomers, also regardless of the type of soil tested. The mini-excavator track, compared to the rubber pad, exhibited more than 137 times greater wear in light soil and more than 234 times greater wear in heavy soil. This is well illustrated by comparing the unit wear values in different soil conditions (Figure 10).

**Figure 11:**
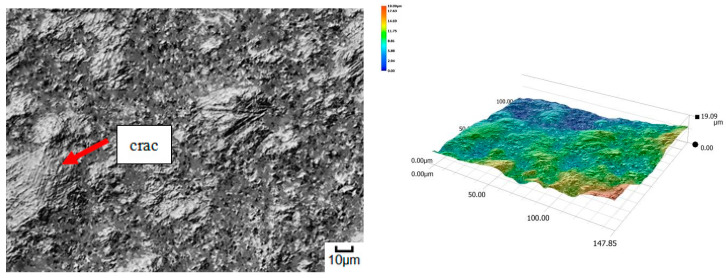
Condition of the friction surface and profile after wear in the light soil of a tractor track 2000×.

**Figure 12:**
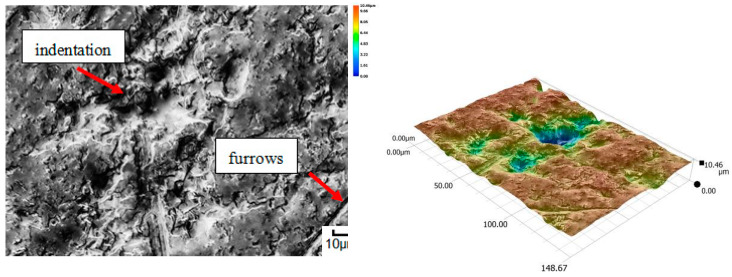
Condition of the friction surface and profile after wear in the light soil of a mini-excavator track 2000×.

**Figure 13:**
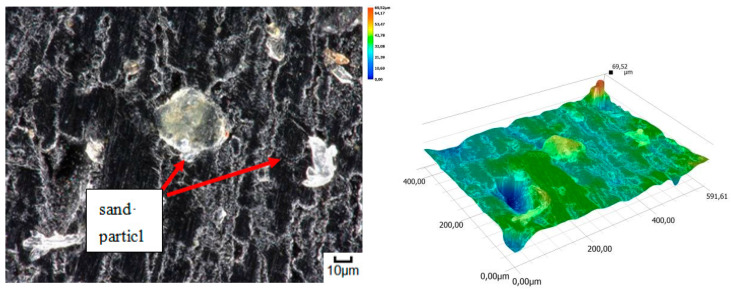
Condition of the friction surface and profile after wear in the light soil of a steel-rubber stave 700×.

**Figure 14:**
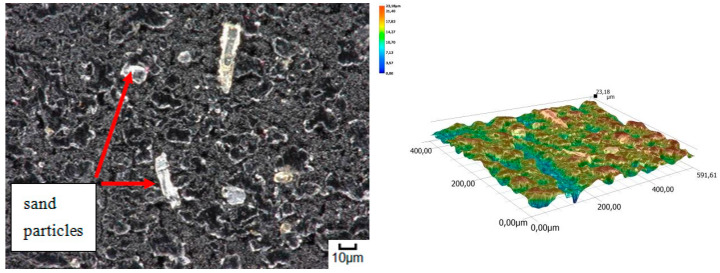
Condition of the friction surface and profile after wear in the light soil of a rubber pad 700×.

**Figure 15:**
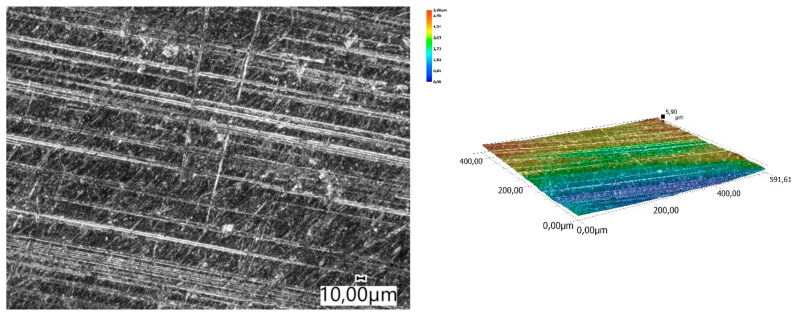
Condition of the friction surface and profile after wear in the light soil of Hadfield cast steel.

The obtained relations can be explained by the wear patterns of the tested materials. For material wear in light soil, loosely bound sand grains (characterised by a high freedom of movement) caused scratching, furrowing of the friction surface, as well as leaving individual sand particles on the surface layer (Figure 11, Figure 12, Figure 13, Figure 14 and Figure 15). This testifies to the point effect of sand grains on the material surface. Fatigue wear, resulting from repeated application of pressures on the friction surface of the rounded SiO_2_ grains (dark areas), dominated on surfaces worn in light soils. The multi-cycle wear consisted of elastic deformation, plastic deformation, micro-volume crushing, structure deformation and shearing of these irregularities. Furrows formed from the cutting action of the sharp sand grain edges are visible. This first phenomenon dominates in the friction process. With the increase of the fine fractions in the soil, they penetrated the surface discontinuities, in a way acting as a protection against the intense impact of the sand fractions. Hence, scratches and sand particle residues occur on the surface worn in heavy soil.

**Figure 16:**
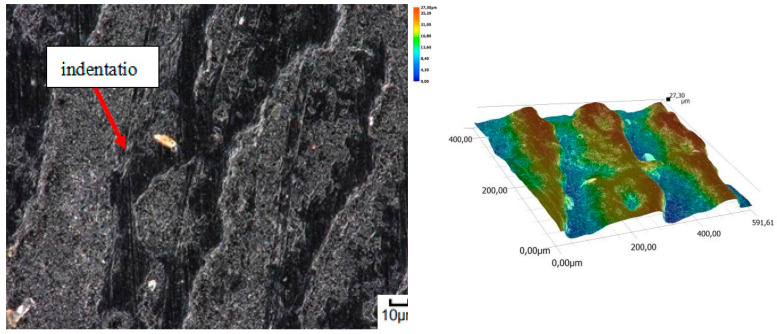
Condition of the friction surface and profile after wear in the heavy soil of a tractor track 700×.

**Figure 17:**
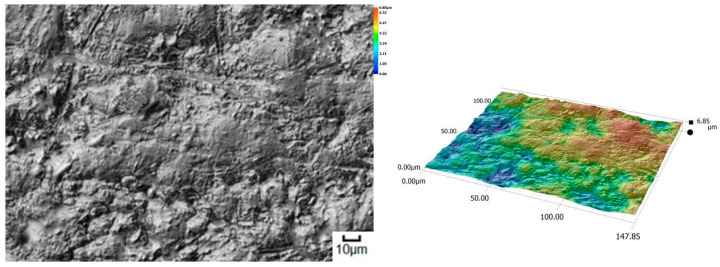
Condition of the friction surface and profile after wear in the heavy soil of a mini-excavator track 2000×.

**Figure 18:**
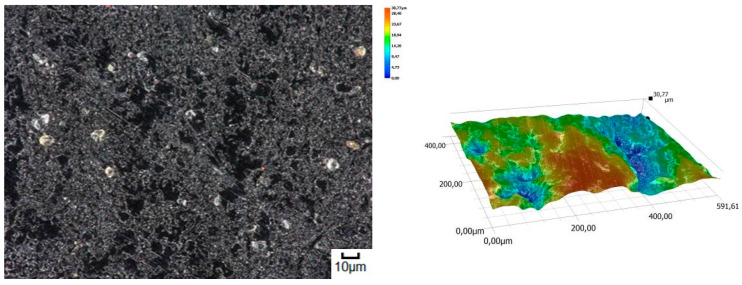
Condition of the friction surface and profile after wear in the heavy soil of a steel-rubber stave 700×.

**Figure 19:**
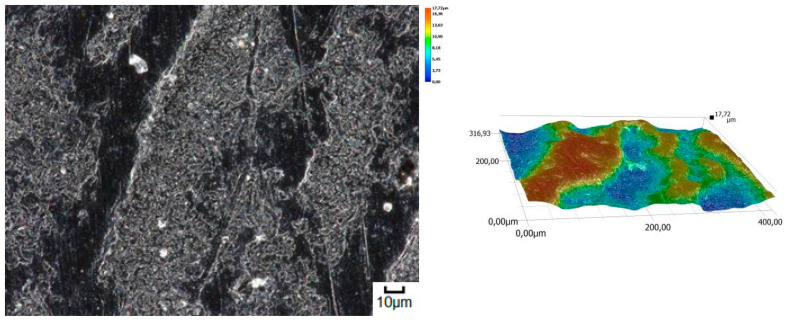
Condition of the friction surface and profile after wear in the heavy soil of a rubber pad 700×.

**Figure 20:**
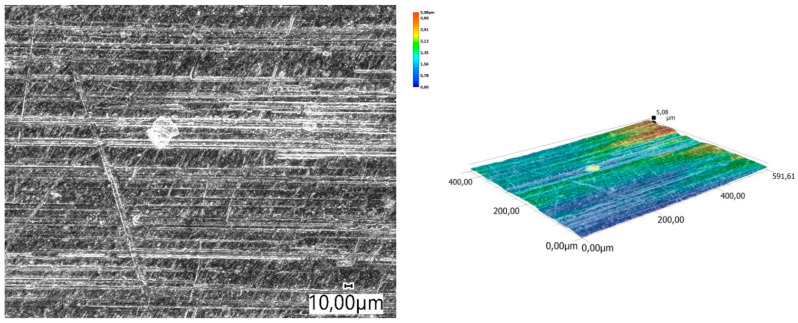
Condition of the friction surface and profile after wear in the heavy soil of Hadfield cast steel.

A different wear pattern can be observed in soils with a higher content of dust and clay particles. Combined with the moisture, the clay in the heavy soil mass forms a binder that holds the abrasive particles together, causing “tears” on the surface layer of the material. Wear with reinforced abrasive grains occurs in this case. The soil mass contacts the material discretely and the intensity of the impact depends on the fixation of the grains in the soil mass. The nature of the wear changes and increases in value. The number of indentations increases, which confirms the different character of wear with a larger friction surface (Figure 16, Figure 17, Figure 18, Figure 19 and Figure 20).

Based on the calculated K_b_ wear resistance index (Table 5), it can be concluded that in both soil types, the wear resistance of the rubber pad was the best of all tested materials compared to Hadfield steel. This material exhibited the highest strength highest (more than 2.4 times) in light soil. The lowest wear strength value (more than 217 times lower) was recorded for the mini-excavator track in heavy soil. The other materials achieved lower abrasive wear strength than Hadfield steel.

## 4. Conclusions and Discussion

The wear properties of the materials accepted for soil abrasive testing depend on the grain size of the soil and the chemical composition of the material. Elastomers containing low-density polyethylene (PE-LD) exhibit lower abrasion wear resistance compared to elastomers containing high-density polyethylene (PE-HD). In light soils, local scratching and furrowing occur due to the action of loosely bound sand particles.

In the heavy abrasive soil, the highest values of the wear intensity of the tested materials were recorded. In the case of the track from the mini-excavator, this value was more than six times higher in relation to the light soil. Loam occurring in heavy soil, in combination with moisture, creates a binder, causing “tearing out” of the surface layer of the tested materials, which increases the number of pits and changes the nature of wear. Due to the fact that heavy soils also contain hard sand particles (approximately 1200 HV), the wear process takes place with hardened abrasive grains.

The best wear-prevention properties, measured using the abrasion resistance index K_b_, were found in a rubber pad composed of high-density polyethylene, low-density polyethylene, ethylene acrylate/ethyl copolymer (ethylene acrylate 18%), and ethylene/propylene copolymer with an ethylene content of 60%. Its strength was more than 2.4 times that of a comparable Hadfield steel. The worst wear parameters were obtained for a mini-excavator track containing low-density polyethylene, high-density polyethylene, ethylene/propylene copolymer with an ethylene content of 60%, oxidised polyethylene, ethylene-acrylic acid, and Na and Zn type ionomers. The K_b_ value for Hadfield steel did not even reach 1%.

Further research should include tests to determine the proportions of the components present in given elastomers.

## Figures and Tables

**Table 1:** Characteristics of the abrasive soil mass.

Granulometric Groups	Fraction Diameter (mm)	Fraction Content (%)	
Sand	2.0–0.05	67.17	16.20
Fines	0.05–0.002	31.03	77.30
Silt	<0.002	1.80	6.50
Determined as per PN–EN ISO 14688–2(2006)		Loamy sand–light soil	Ordinary soil–heavy soil

**Table 2:** Chemical Composition of Hadfield Steel.

C	Mn	Cr	Ni	S	D	Si
**(% mass)**						
1.27	13.15	0.67	0.42	0.03	0.07	0.44

**Table 3:** Properties of Materials Accepted for Testing.

	Hardness	Density (g/cm^3^)
Track from a tractor	72 Shore scale A	1.1
Track from a mini excavator	66 Shore scale A	1.1
Steel-rubber stave	73 Shore scale A	2.3
Rubber pad	70 Shore scale A	1.2
Hadfield cast steel	HV 281	7.2

**Table 4:** Average mass wear of the tested materials.

Mass Wear [g]				
Material	Light Soil	Standard Deviation	Heavy Soil	Standard Deviation
Track from a tractor	0.2313	0.0847	0.4661	0.1821
Track from a mini excavator	0.4797	0.1812	2.9085	1.0919
Steel-rubber stave	0.0315	0.0119	0.0391	0.0145
Rubber pad	0.0035	0.0013	0.0122	0.0047
Hadfield cast steel	0.0514	0.0197	0.0897	0.0339

**Table 5:** Summary of wear strength.

	Light Soil		Heavy Soil	
	K_b_	Confidence Interval	K_b_	Confidence Interval
Track from a tractor	0.0339	0.0211	0.0294	0.0110
Track from a mini excavator	0.0163	0.0218	0.0047	0.3273
Steel-rubber stave	0.5212	0.0047	0.7328	0.0020
Rubber pad	2.4476	0.0010	1.2254	0.0018
Hadfield cast steel	1.0000	0.0086	1.0000	0.0148

## Data Availability

The data is with the authors.
